# Diagnostic accuracy of ^18^F amyloid PET tracers for the diagnosis of Alzheimer’s disease: a systematic review and meta-analysis

**DOI:** 10.1007/s00259-015-3228-x

**Published:** 2015-11-28

**Authors:** Elizabeth Morris, Anastasia Chalkidou, Alexander Hammers, Janet Peacock, Jennifer Summers, Stephen Keevil

**Affiliations:** King’s Technology Evaluation Centre, King’s College London, St Thomas’ Hospital, Westminster Bridge Road, London, SE1 7EH UK; Department of Biomedical Engineering, Division of Imaging Sciences and Biomedical Engineering, King’s College London, St Thomas’ Hospital, London, UK; Division of Health and Social Care Research, King’s College London, London, UK; NIHR Biomedical Research Centre at Guy’s and St Thomas’ NHS Foundation Trust, King’s College London, London, UK; Department of Medical Physics, Guy’s and St Thomas’ NHS Foundation Trust, St Thomas’ Hospital, London, UK

**Keywords:** Florbetapir, Florbetaben, Flutemetamol, Amyloid, PET, Alzheimer’s disease

## Abstract

Imaging or tissue biomarker evidence has been introduced into the core diagnostic pathway for Alzheimer’s disease (AD). PET using ^18^F-labelled beta-amyloid PET tracers has shown promise for the early diagnosis of AD. However, most studies included only small numbers of participants and no consensus has been reached as to which radiotracer has the highest diagnostic accuracy. First, we performed a systematic review of the literature published between 1990 and 2014 for studies exploring the diagnostic accuracy of florbetaben, florbetapir and flutemetamol in AD. The included studies were analysed using the QUADAS assessment of methodological quality. A meta-analysis of the sensitivity and specificity reported within each study was performed. Pooled values were calculated for each radiotracer and for visual or quantitative analysis by population included. The systematic review identified nine studies eligible for inclusion. There were limited variations in the methods between studies reporting the same radiotracer. The meta-analysis results showed that pooled sensitivity and specificity values were in general high for all tracers. This was confirmed by calculating likelihood ratios. A patient with a positive ratio is much more likely to have AD than a patient with a negative ratio, and vice versa. However, specificity was higher when only patients with AD were compared with healthy controls. This systematic review and meta-analysis found no marked differences in the diagnostic accuracy of the three beta-amyloid radiotracers. All tracers perform better when used to discriminate between patients with AD and healthy controls. The sensitivity and specificity for quantitative and visual analysis are comparable to those of other imaging or biomarker techniques used to diagnose AD. Further research is required to identify the combination of tests that provides the highest sensitivity and specificity, and to identify the most suitable position for the tracer in the clinical pathway.

**Electronic supplementary material:**

The online version of this article (doi:10.1007/s00259-015-3228-x) contains supplementary material, which is available to authorized users.

## Introduction

The 2007 International Working Group (IWG) for New Research Criteria for the Diagnosis of Alzheimer’s disease marked a conceptual change. The traditional view of Alzheimer’s disease (AD) as a purely clinicopathological entity changed to one of AD as a clinicobiological entity. This in turn led to the introduction of imaging or tissue biomarker evidence into the core diagnostic pathway [1]. The inclusion of this evidence enabled diagnosis to be extended to earlier stages of AD, opening the way for the development of therapies earlier in the course of the disease when clinical symptoms are absent [2]. In recent years there has been increasing interest in the use of beta-amyloid PET radiotracers for the diagnosis of AD.

The clinical features of AD include amnesic memory impairment, language deterioration and visuospatial deficits, as well as functional and behavioural changes [1, 3]. The original criteria for diagnosing AD were established in the autumn of 1983 [2, 4]. These criteria, commonly referred to as the NINCDS-ADRDA criteria, have been used for almost 30 years, provide a sensitivity of 81 % and a specificity of 70 % [3, 5] and are widely used in clinical trials and in clinical research. According to these criteria the diagnosis of AD is categorized as probable, possible, and definite [2, 4]. Although the diagnosis of probable and possible AD can be established clinically, a definite diagnosis requires histopathological confirmation [2, 4]. These criteria were revised in 2011 to include core clinical criteria for probable and possible (known as mild cognitive impairment, MCI, in the new version) AD, and the rationale for including imaging and tissue biomarkers of the pathophysiological process of AD in the diagnostic criteria was outlined. These biomarkers were divided into two classes on a biological basis: biomarkers of brain amyloid-beta protein deposition and positive PET amyloid imaging [4, 6].

In the literature, there are multiple potential diagnostic radiotracers at different stages of development. Pittsburgh Compound B (PiB) is a modification of thioflavin-T, a histological dye used to bind to amyloid plaques in vitro [7]. ^11^C-labelled PiB has been shown to cross the blood–brain barrier and bind to amyloid plaques with high affinity in vivo in an animal study [7], and studies in human subjects have demonstrated its ability to distinguish between patients with AD and healthy controls (HC) [8, 9]. ^11^C-PiB may also be beneficial in identifying patients in whom MCI will progress to AD [10, 11] and two case studies have demonstrated a relationship between ^11^C-PiB retention and post-mortem pathological findings [12, 13]. However, due to the 20-min half-life of ^11^C, ^11^C-PiB can only be used in large PET centres with their own on-site cyclotron and radiopharmacy facilities.

^18^F is a more suitable radioisotope for widespread clinical use as its longer half-life of 110 min allows distribution from a production site to multiple PET centres. Three ^18^F-labelled tracers have been developed which are starting to be used clinically. Flutemetamol (GE Healthcare) is a close structural analogue of ^11^C-PiB, whilst florbetapir (Amyvid, Eli Lilly) and florbetaben (Neuraceq, Piramal Imaging Limited) are derived from stilbene [14]. Marketing authorizations were granted by the European Medicines Agency for florbetapir in 2013 [15], and for florbetaben and flutemetamol in 2014 [16, 17].

We performed a systematic review of published studies that explored the diagnostic accuracy of the amyloid tracers which have European marketing authorization. For the purposes of defining the comparator, we assumed that standard care includes clinical diagnosis or histopathology results. We also investigated the quality of the available studies, compared their technical characteristics and performed a meta-analysis to examine the investigated tracer’s sensitivity and specificity for detecting AD.

## Methods

### Study identification and selection

The criteria for including studies were as follows:

- Includes patients with a diagnosis of AD and a control group
- Analysis includes more than ten patients
- Investigation of the diagnostic accuracy of florbetapir, florbetaben or flutemetamol PET uptake compared to clinical diagnosis or histopathology
- Publication of full paper in a peer-reviewed scientific journal

### Search methods

The literature search was carried out using MEDLINE (1946 – week 25 2014; Ovid interface), EMBASE (1947 – week 25 2014; Ovid interface) and the Cochrane Library for relevant studies published between January 1990 and June 2014. MEDLINE was additionally searched using the PubMed interface using the tracer name only. Terms designed to identify the disease, PET tracers and diagnostic accuracy were used within the search strategy. Supplementary Table S1 shows the full electronic search strategy used for MEDLINE. The search was restricted to studies in humans and papers presented in English. Full text publications were obtained and reviewed. When multiple publications presented results using the same patient cohort, the most recent or the most complete publication was selected for inclusion. Review articles and references from accepted articles were searched for any additional papers.

### Data extraction and management

Details of the included studies are presented in Tables 1, 2 and 3. The data collected included the overall study characteristics, technical details of the PET acquisition and the characteristics relevant to the diagnostic accuracy of the PET scans. The data relevant to diagnostic accuracy are presented in Table 4. Microsoft Excel software was used for data collection and management.

**Table 1:** Procedures used in the included studies

Reference	Radiopharmaceutical	Dose (MBq)	Reference tests	MRI	PET scanner	Immobilization devices	Uptake period (min)	Scan duration (min)
[18]	Florbetaben	300 ± 60	NINCDS-ADRDA criteria, CDR, CERAD test battery, other cognitive tests	Yes	Stand-alone PET or PET/CT scanner, models not specified	Yes	90	20
[19]	Florbetapir	4/kg	NINCDS-ADRDA criteria, MMSE, FCSRT, other cognitive tests	Yes	Philips Dual Gemini, GE Discovery RX VCT 64, Siemens Biograph 6 TruePoint HiRez	NA	50	10
[20]	Florbetapir	370	Post-mortem analysis using modified CERAD criteria	No	Stand-alone PET or PET/CT scanner, models not specified	NA	50	10
[14]	Flutemetamol	197 ± 5.9	NIA-AA criteria, MMSE, CDR, other cognitive tests	Yes	Siemens ECAT ACCEL scanner	Yes	85	30
[21]	Florbetapir	365	NINCDS-ADRDA criteria, MMSE, CDR, other cognitive tests	Yes	Siemens Biograph mCT PET/CT	NA	50	10
[22]	Florbetapir	370	NINCDS-ADRDA criteria, MMSE	No	Not specified	Yes	50	10
[23]	Florbetapir	370	NINCDS-ADRDA criteria, MMSE, demographic information	No	Siemens Biograph 2-slice PET/CT, Siemens Biograph mCT 40-slice PET/CT, Siemens ECAT HR+	NA	50	10
[24]	Flutemetamol	120^a^ or 185	NINCDS-ADRDA criteria, MMSE, CDR, other cognitive tests	Yes	Siemens Biograph 16-slice PET/CT, Siemens ECAT EXACT HR+ scanner, GE Advance scanner	NA	85	30
[25]	Florbetaben	300	NINCDS-ADRDA criteria, MMSE, CRS, other cognitive tests	Yes	Philips Allegro	Yes	90	20

*CDR* Clinical Dementia Rating, *CERAD* Consortium to Establish a Registry for Alzheimer’s disease, *FCRST* Free and Cued Selective Reminding Test, *MMSE* Mini-Mental State Examination, *NA* not available, *NIA-AA* National Institute on Aging – Alzheimer’s Association, *NINCDS-ADRDA* National Institute of Neurological and Communicative Disorders and Stroke-Alzheimer’s Disease and Related Association

^a^Subjects who underwent two scans received a lower dose of 120 MBq

**Table 2:** Analyses used in the included studies

Reference	Reconstruction method	PET corrections mentioned						Qualitative assessment	Quantitative assessment	Reference region	Template
		Attenuation	Scatter	Random	Decay	Dead time	Motion				
[18]	Iterative	Yes	Yes	No	Yes	Yes	No	Three-grade scale/majority	SUVr	Cerebellar cortex	MNI MRI singles-subject brain template using SPM
[19]	Iterative	Yes	Yes	Yes	Yes	No	Yes	Binary scale/agreement	SUVr	Whole cerebellum	Talairach space template using PMOD
[20]	Iterative	No	No	No	No	No	No	Binary scale/majority	SUVr	Whole cerebellum	Talairach space template using SPM
[14]	Iterative	No	No	No	Yes	No	Yes	Binary scale/agreement	SUVr	Cerebellar cortex	Unclear
[21]	Iterative	Yes	Yes	Yes	No	No	No	None	SUVr	Whole cerebellum	MNI T1 template using SPM
[22]	Iterative	Yes	No	No	No	No	No	Binary scale/agreement	Not included	Cerebellum	No
[23]	Iterative	No	No	No	No	No	No	Binary scale/majority	Not included	Cerebellum/occipital cortex	AV-133 PET template
[24]	Filtered back projection or iterative	No	No	No	No	No	No	Binary scale/agreement	SUVr	Cerebellar cortex	MNI space
[25]	Iterative	Yes	No	No	No	No	No	Three-grade scale/unspecified	SUVr	Cerebellar cortex	Unclear

*MNI* Montreal Neurological Institute, *SUVr* standardized uptake value ratio, *SPM* statistical parametric mapping

**Table 3:** Clinical characteristics of the patients and healthy controls in the included studies

Reference	Subjects per group (*n*)	Mean age (years)	Cognitive tests			Final diagnosis
			AD	MCI	Controls	
[18]	AD: 78 MCI: 0 Controls: 68	AD: 70.7 MCI: 0 Controls: 68.2	MMSE: 22.6 (2.3) CDR: 0: 0, 0.5: 36 %, 1.0: 60 %, 2.0: 4 %	NA	MMSE: 29.3 (0.8) CDR: 0: 100 %, 0.5: 0 %, 1.0: 0 %, 2.0: 0 %	Clinical diagnosis – NOS
[19]	AD: 13 MCI: 12 Controls: 21	AD: 67.8 MCI: 75 Controls: 66.2	MMSE: 23 (3.6)	MMSE: 25.9 (2.9)	MMSE: 29 (1.3)	Clinical diagnosis – NOS
[20]	AD: 29 MCI: 5 Other dementia: 13 Controls: 12	AD: NS Other dementia: NS Controls: NS	NA	NA	NA	Autopsy
[14]	AD: 36 MCI: 68 Controls: older 41, transitional 10, young 11	AD: 76.8 MCI: 71.9 Controls: older 66.9, transitional 50.8, young 39.5	MMSE: 19.4 (4.6) Global CDR: 0.9 ± 0.4	MMSE: 27.4 (1.7) Global CDR: 0.5	MMSE: older 29.4 (0.6), transitional 29.8 (0.6), young 29.2 (0.9) Global CDR: 0	Clinical diagnosis – cross-sectional
[21]	AD: 12 MCI: 13 Controls: 11	AD: 75.8 MCI: 70.0 Controls: 69.3	MMSE: 12.9 (3.7) Global CDR: 1.1 ± 0.3	MMSE: 19.7 (2.4) Global CDR: 0.5 ± 0.0	MMSE: 27.9 (1.6) Global CDR: 0.0 ± 0.0	Clinical diagnosis – NOS
[22]	AD: 19 MCI: 0 Controls: 21	AD: 73 Controls: 67	MMSE: 20 (7)	NA	MMSE: 29 (1)	Clinical diagnosis – NOS
[23]	AD: 10 Controls: 5	AD: 75.7 Other: 68.2/70.18 Controls: NA	MMSE: 20.1 (3.57)	NA	MMSE: 29.6 (0.55)	Clinical diagnosis – cross-sectional
[24]	AD: 27 MCI: 20 Controls: 15/10	AD: 69.6 MCI: 72.7 Controls: 68.7/37.9	MMSE: 23.3 (2.18) Global CDR: NA	MMSE: 28.0 (0.94) Global CDR: NA	MMSE: 29.4 (0.97) Global CDR: NA	Clinical diagnosis – cross-sectional
[25]	AD: 30 MCI: 20 Controls: 32	AD: 72.0 MCI: 73.4 Controls: 70.7	MMSE: 22.8 (3.7) Global CDR: NA	MMSE: 27.4 (1.9) Global CDR: NA	MMSE: 29.6 (0.7) Global CDR: NA	Clinical diagnosis – cross-sectional

MMSE scores presented as means (standard deviation)

*AD* Alzheimer’s disease, *MCI* mild cognitive impairment, *MMSE* Mini-Mental State Examination, *CDR* Clinical Dementia Rating, *NA* not available, *NOS* not otherwise specified

**Table 4:** Outcome data for meta-analysis

Reference	Type of analysis	Radiotracer	Study groups	No. of patients						
				AD	HC	Others included	AD		Non-AD	
							With positive PET	With negative PET	With positive PET	With negative PET
[18]	Visual	Florbetaben	HC vs. AD	78	68	0	62	16	6	62
	Quantitative	Florbetaben	HC vs. AD	78	68	0	66	12	6	62
[19]	Visual	Florbetapir	HC vs. AD	13	21	0	11	2	13	8
	Visual	Florbetapir	HC vs. MCI vs. AD	13	21	12 MCI	11	2	19	14
	Quantitative	Florbetapir	HC vs. MCI vs. AD	13	21	12 MCI	12	1	3	30
[20]	Visual	Florbetapir	HC vs. MCI vs. AD	29	12	5 MCI, 13 OD	36	3	0	20
	Quantitative	Florbetapir	HC vs. MCI vs. AD	29	12	5 MCI, 13 OD	38	1	0	20
[14]	Visual	Flutemetamol	HC vs. AD	36	41	0	35	1	6	35
			HC vs. MCI vs. AD	36	41	68 MCI	35	1	35	74
	Quantitative	Flutemetamol	HC vs. AD	36	41	0	34	2	7	34
			HC vs. MCI vs. AD	36	41	68 MCI	34	2	35	74
[21]	Quantitative	Florbetapir	HC vs. AD	12	11	13 MCI	11	1	9	15
[22]	Visual	Florbetapir	HC vs. AD	19	21	0	18	1	1	20
[23]	Visual	Florbetapir	HC vs. AD	10	5	0	9	1	0	5
[24]	Visual	Flutemetamol	HC vs. AD	27	15	0	25	2	1	14
			HC vs. MCI vs. AD	27	15	20 MCI	25	2	10	25
	Quantitative	Flutemetamol	HC vs. AD	27	15	0	25	2	1	14
			HC vs. MCI vs. AD	27	15	20 MCI	25	2	11	24
[25]	Visual	Florbetaben	HC vs. AD	30	32	0	29	1	5	27
			HC vs. MCI vs. AD	30	32	20 MCI	29	1	17	35
	Quantitative	Florbetaben	HC vs. AD	30	32	0	29	1	5	27

*HC* healthy control, *AD* Alzheimer’s disease, *MCI* mild cognitive Impairment, *OD* other dementia

The data fields to extract from the publications were chosen according to two criteria: the information required to perform quantitative analysis on the data, and those required to replicate the study design, as described in the modified Quality Assessment of Diagnostic Accuracy Studies (QUADAS) tool [26].

### QUADAS assessment of methodological quality

The QUADAS tool was developed and evaluated by Whiting et al. [26, 27] and is recommended by the Cochrane diagnostic accuracy systematic reviews [28] to provide a methodological assessment of the quality of studies of diagnostic accuracy. The tool comprises 14 questions designed to identify potential areas of bias within a study, and can be tailored to suit the design of the included studies. The original and a modified QUADAS list for PET radiotracers [29] were reviewed by two investigators (A.C., A.H.) and a consensus was agreed regarding the guidelines for scoring each item (Supplementary Table S1). The ‘reference standard’ was either clinical diagnosis or histopathology, and the ‘index test’ was the PET scan with the radiotracer used in the publication in question. Two of the questions were not relevant, and were removed from the list. Each item in the list was scored as ‘yes’ if all aspects of the criteria were fulfilled, ‘no’ if there were aspects of the criteria missing, and ‘unclear’ if it was not possible to conclude either way from the evidence presented in the publication. Two assessors (E.M., A.C.) independently reviewed and scored the selected publications. Any disagreements were resolved by discussion and the final scores represent the consensus. The meta-analysis was performed independently of the results of the QUADAS assessment, as the QUADAS tool was not designed to weight data for this purpose [30, 31].

### Meta-analysis

The included studies were reviewed and outcome data were extracted for the meta-analysis. Given the small study sample size coupled with large proportions, assumptions of normality were not justified as the proportions were negatively skewed. Therefore, the logit transform of one minus each proportion (i.e. log[(1 − *p*)/*p*]) was used in the meta-analyses as a normalizing transformation. The results were then back-transformed to the natural scale for presentation. A test of statistical heterogeneity was performed for each meta-analysis and a random effects model was fitted. Numerator values of ‘0’ (only in specificity) were replaced with ‘0.5’. Pooled values were calculated for all radiotracers and each separately based on subgroup analysis. Subgroups were defined based on (a) study population (AD vs. HC and/or MCI), and (b) the method used for assessing PET uptake (visual interpretation vs. SUV-based quantification). All meta-analyses were conducted using the Metan procedure in Stata v. 11.0.

## Results

### Study identification and selection

**Fig. 1:**
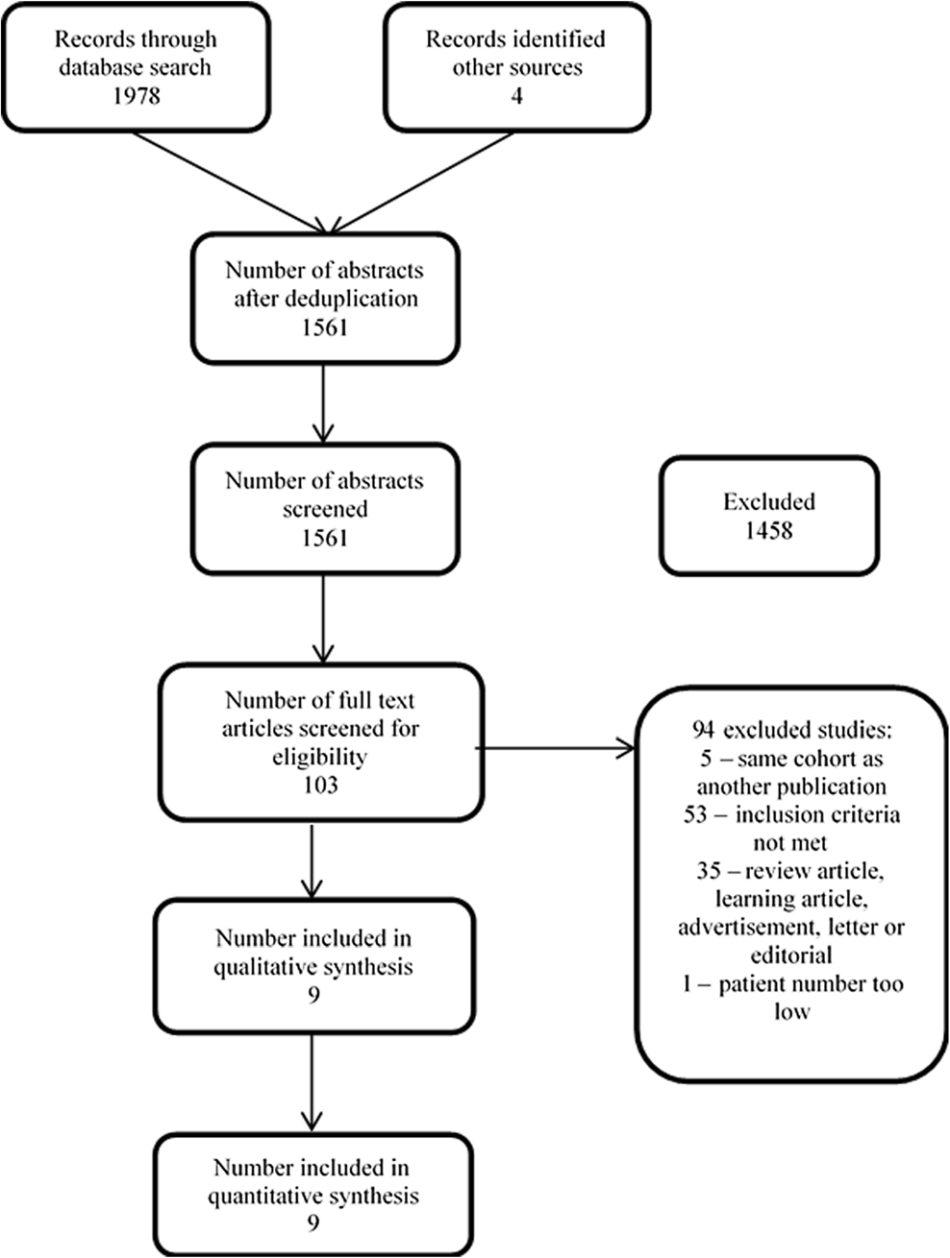
Flow chart of study selection

The search conducted in EMBASE and MEDLINE returned 1,978 references and the Cochrane library returned five. Two additional articles were returned from PubMed, and two from the reference list of Newberg et al. [22]. The total number of abstracts after removal of duplicates was 1,561. These abstracts were screened using the inclusion criteria and 103 articles were considered eligible to be read in full. Nine articles were considered eligible for inclusion [14, 18–25] and the remaining 92 articles were excluded; the reasons for exclusion are given in Fig. 1. Figure 1 presents the data using a four-phase flow diagram as recommended in the PRISMA statement [32].

### Study characteristics

Technical details of the included studies are presented in Tables 1 and 2. Five of the included studies investigated florbetapir [19–23], two investigated florbetaben [18, 25] and two investigated flutemetamol [14, 24]. In total, there were 662 participants in the included studies, with a median number per study of 59 (interquartile range 40–62). The characteristics of the PET scans, such as administered dose, uptake period and scan duration, were similar among studies using the same radiotracer. However, most studies did not provide information regarding the specific reconstruction algorithms used. There was limited but varied information provided on corrections made to the data prior to reconstruction.

The methods for reporting the PET scans were similar among the nine studies with eight reporting qualitative results, and seven quantitative. Six of the studies used a binary system to analyse the qualitative results, with two studies using the three-point system negative, minor and significant amyloid presence (Table 2). All studies reported the use of the standardized uptake value ratio (SUVr), in five using as a reference the whole cerebellum and in four the cerebellar cortex (Table 2), a simple semiquantitative analysis method [33]. Only three studies used a predefined cut-off value for determining whether the SUVr was amyloid-positive or amyloid-negative. In the remaining studies, the cut-off value was calculated using the optimum cut-off approach [34, 35].

All studies except two [5, 20] used clinical examination to establish the diagnosis and all reported the results of the Mini-Mental State Examination (MMSE). In the majority of studies the mean MMSE score for AD patients was approximately 23 [6–9, 18, 19, 24, 25] and in only one study was the mean MMSE score below 18 [10, 20], indicating that the majority of the included patients clinically had MCI as classified by the MMSE (Table 3).

### QUADAS assessment of methodological quality

**Fig. 2:**
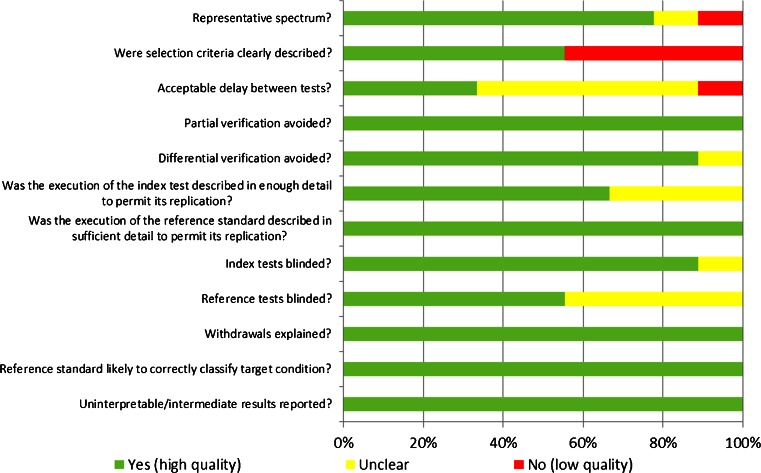
Methodological quality

Each of the included studies was ranked according to the QUADAS description provided in Supplementary Table 1. The results are presented in Fig. 2. Two studies scored ‘yes’ for all 12 items [18, 25]. All nine studies scored ‘yes’ for five items (questions 4, 7, 11, 12 and 14). The QUADAS items scoring lowest were 2, 3 and 9.

The inclusion and exclusion criteria (item 2) were clearly described in most studies. Where studies received an ‘unclear’ ranking, this was due to the omission of the recruitment period. The delay between the reference and index tests (item 3) was specified as less than 4 weeks in only three of the studies. One of the remaining studies specified a delay longer than 4 weeks, and the rest were unclear on the timing between the two. Items 6 and 7 refer to the adequacy of the description provided by the authors to allow replication of the index and reference test, respectively. Generally, the description of the index test was adequate, but three studies made no mention of immobilization devices or motion correction. For item 7, eight studies [14, 18, 19, 22, 24, 25] provided references to standard clinical diagnosis tests, and one study [20] provided details of the post-mortem method. All studies provided specific details about withdrawals, or the results were presented for the same number of participants as originally entered the study.

### Meta-analysis

The pooled estimates with 95 % confidence intervals (CI) for sensitivity, specificity, and positive and negative likelihood ratios are presented in Table 5. The results for sensitivity by all subgroups ranged from 89 % to 97 %, while for specificity the values ranged more widely, from 63 % to 93 %. The values for both sensitivity and specificity were similar for both visual and quantitative analysis. Overall, sensitivity was higher than specificity for all subgroups, except for the visual/florbetaben/HC-vs.-AD subgroup, which had similar values for sensitivity (89 %, 95 % CI 55–98 %) and specificity (89 %, 95 % CI 81–94 %). The negative likelihood ratios were all smaller than 0.2 and some were <0.1 indicating almost no likelihood of AD. In contrast, there was great variability in the positive likelihood ratios with the lowest value for florbetapir in a mixed population (1.5) and using visual analysis, and the highest value for florbetapir in a mixed population but using quantitative analysis (10.2). However, the wide CIs obtained mean that it is difficult to select the best method of analysis with certainty.

**Table 5:** Pooled estimates with 95 % confidence interval for sensitivity, specificity, and positive and negative likelihood ratios

Type of analysis	Radiopharmaceutical	Study groups	No. of studies	Sensitivity (95 % CI) (%)	Specificity (95 % CI) (%)	Likelihood ratio (95 % CI)	
						Positive	Negative
Visual	Florbetaben	HC vs. AD	2	89 (55–98)	89 (81–94)	7.5 (4.3–13.2)	0.12 (0.02–0.62)
		HC vs. MCI vs. AD	1	97 (79–99.5)	67 (54–79)	3.0 (2.0–4.4)	0.05 (0.01–0.34)
	Florbetapir	HC vs. AD	3	90 (75–96)	81 (24–98)	4.4 (0.3–59.4)	0.17 (0.06–0.44)
		HC vs. MCI vs. AD	2	90 (78–96)	81 (8–99.5)	1.5 (1.0–2.1)	0.16 (0.03–0.72)
	Flutemetamol	HC vs. AD	2	95 (85–98)	87 (75–94)	7.3 (3.7–14.6)	0.06 (0.02–0.18)
		HC vs. MCI vs. AD	2	95 (85–98)	69 (61–76)	3.1 (2.4–3.9)	0.08 (0.03–0.23)
Visual	All	HC vs. AD	7	90 (82–95)	85 (68–94)	6.1 (2.4–15.6)	0.12 (0.06–0.24)
	All	HC vs. MCI vs. AD	5	93 (87–96)	66 (52–77)	2.5 (1.8–3.7)	0.10 (0.06–0.20)
Quantitative	Florbetaben	HC vs. AD	2	91 (67–98)	87 (81–94)	7.8 (4.6–13.6)	0.15 (0.09–0.25)
		HC vs. MCI vs. AD	0	NA	NA	NA	NA
	Florbetapir	HC vs. AD	1	92 (59–99)	63 (42–79)	2.4 (1.4–4.2)	0.13 (0.02–0.89)
		HC vs. MCI vs. AD	2	96 (84–99)	93 (81–97)	10.2 (3.4–30.2)	0.05 (0.01–0.18)
	Flutemetamol	HC vs. AD	2	94 (84–98)	85 (73–92)	6.1 (3.2–11.6)	0.07 (0.03–0.19)
		HC vs. MCI vs. AD	2	94 (84–98)	68 (60–75)	2.9 (2.3–3.8)	0.09 (0.04–0.24)
Quantitative	All	HC vs. AD	5	90 (83–94)	84 (71–91)	5.5 (3.1–9.9)	0.13 (0.08–0.20)
	All	HC vs. MCI vs. AD	4	94 (88–97)	79 (62–89)	3.5 (2.2–5.6)	0.07 (0.03–0.16)

*HC* healthy control, *AD* Alzheimer’s disease, *MCI* mild cognitive Impairment, *NA* not available

## Discussion

This systematic review was designed to investigate the accuracy of three new radiotracers in the diagnosis of AD by their ability to identify amyloid beta plaques in vivo. The objectives were to investigate the quality of available studies, compare the technical characteristics and perform a meta-analysis to examine the sensitivity and specificity of the included tracers. In general, the methodological quality assessed using QUADAS characteristics was good. Many of the omissions were related to inadequacies of reporting rather than deficiencies in trial design. Where the reference test design scored badly, this was often related to a lack of information on patient immobilization or motion correction techniques. Patients with AD may be less able to follow instructions, such as to remain still during the scan [36]. Head motion during a PET scan can affect both visual interpretation and quantitative analysis [36]; therefore, the inclusion of appropriate head fixation devices or motion correction methods is an important aspect of study design.

The PET imaging protocol can be a source of variability in the results especially when quantitative analysis is performed [37]. Whilst previous systematic reviews and meta-analyses with non-FDG radiotracers identified large variations in scanning protocols [29, 38], technical characteristics were generally comparable between the included studies. The administered dose, uptake period and scan duration were identical across all studies of the same radiotracer. The technical similarity of the studies can be partially attributed to the involvement of the manufacturers as sponsors [14, 20, 23, 24] or training providers [19], or the adoption of published protocols by the authors [21, 22]. The scanner models varied among centres, or were not specified, which would be expected for radiotracers used in clinical practice.

An important concept in both visual and quantitative amyloid PET interpretation is that of amyloid positivity. Although differences between studies exist, in both visual and quantitative assessment, positivity was defined based on the presence or absence of tracer uptake in the brain cortical regions in relation to a reference region believed not to accumulate amyloid, most commonly the cerebellum. A negative scan will display a clear image of the corpus callosum and pons in a midline sagittal slice, and transverse slices will display normal white matter patterns [11, 39]. Quantitative analysis has been advocated over visual interpretation in patients in whom the detection of small amounts of amyloid beta in early disease stages is needed as well as for monitoring the effect of amyloid beta-cleaving drugs [6, 18]. The reason for the latter is that treatments intended to remove amyloid beta plaques may have modest effects on the amyloid PET signal that are not apparent by visual comparison of scans [12, 40]. It should be noted that visual analysis is usually performed using a binary scale while quantitative analysis usually involves receiver operating characteristic analysis without prespecified cut-off values in most cases. As a result these data will be subject to over-fitting possibly resulting in sensitivity and specificity values that are overly optimistic [13, 35]. On the other hand, visual interpretation depends on the observer’s experience and lacks a clear cut-off value between normal and pathological findings. It should be noted that in almost all cases of visual assessment, multiple readers had to reach agreement for a scan to be classified as positive or negative. This is contrary to everyday clinical practice and will have an effect on diagnostic accuracy.

Most studies used similar visual and quantitative analysis methods with variations on the use of an atlas or template for identifying regions of the brain. The type of atlas used affects the anatomical accuracy, in particular whether a single-subject atlas is used, or one derived from multiple subjects [41]. Approximately half the studies used the cerebellum as the reference region and half the cerebellar cortex for calculating SUVr, and in most cases a binary yes/no answer was used for the visual analysis. The cerebellum is considered an appropriate reference region as post-mortem histopathology has shown low levels of amyloid plaques in this area [42].

The meta-analysis of diagnostic accuracy provided 15 additional pooled outcome estimates with 95 % CIs. The results suggest no noticeable differences in sensitivity and specificity among the different agents. This may be a result of the small sample sizes and wide CIs. Sensitivity values were higher for most subgroups, while specificity was, on average, lower. The sensitivity and specificity results for visual and quantitative analyses were very similar. For both types of analysis, the inclusion of participants with MCI generally improved the sensitivity of the test but worsened the specificity. The use of a combination of both analysis methods has not been tested. The specificity of a test will also be affected by the age of the study subjects as the accumulation of amyloid deposits increases with age without necessarily affecting cognitive function [43]. Studies recruiting younger HC (<60 years old) [14, 24] will show increased specificity as the percentage of amyloid positivity in this population is almost 0 % [43]. As there were no significant differences among the different radiotracers included, no specific radiotracer can be recommended based on the results of this analysis.

The gold standard method for definitive diagnosis of AD is histopathological analysis of post-mortem brain tissue. Clinical diagnostic methods, such as the MMSE and the Clinical Dementia Rating, which allow a diagnosis of probable AD, have a varied sensitivity and specificity. A meta-analysis of the MMSE concluded that this test has modest accuracy, and is most appropriate for ruling out dementia in a primary care setting. The MMSE was able to correctly distinguish patients with dementia from healthy subjects with a pooled sensitivity of 76.1 % (95 % CI 75.3–77.9 %) and a pooled specificity of 88.6 % (95 % CI 87.5–89.6 %) [44]. The aim of amyloid tracers is to provide a method to identify amyloid plaques in vivo, where previously this has only been possible post mortem, and allow physicians to diagnose AD with greater accuracy early in the disease course. The low sensitivity and specificity obtained with clinical diagnostic methods could cause false-positive and false-negative findings in studies that use such methods as the reference standard. However, studies using post-mortem analysis also have drawbacks, such as the length of time between the index test and the post-mortem examination, and the associated procedural cost [20].

There are a variety of alternative imaging and biomarker techniques that can be used to provide complementary information to improve the accuracy in the diagnosis of AD. Two meta-analyses calculated the diagnostic accuracy of alternative neuroimaging techniques (MRI, CT, SPECT, and FDG and ^11^C-PiB PET) and cerebrospinal fluid (CSF) biomarkers CSF Aβ_1–42_, CSF T_tau_ and CSF P_tau_) [43, 45]. The results are presented in Table 6 in comparison with those calculated in this meta-analysis for the AD vs. HC group. With the exception of ^18^F-FDG and ^11^C-PiB PET, the sensitivity of amyloid PET imaging is generally higher for both visual and quantitative analysis methods than with other biomarkers and imaging modalities. The specificity of quantitative analysis of amyloid PET imaging is comparable to that of other methods. However, it should be noted that in the meta-analysis by Bloudek et al. [45] the results from a wide range of tests were pooled; for example, studies with HMPAO, IMP and ECD SPECT were combined in the SPECT category, without addressing their technical and methodological aspects.

**Table 6:** Pooled sensitivity and specificity of other biomarkers and imaging modalities for distinguishing patients with AD from healthy controls from meta-analyses [43, 45] in comparison with our results

Reference		Diagnostic method	Sensitivity (%)		Specificity (%)	
			Pooled value	95 % CI	Pooled value	95 % CI
[45]		MRI	83	77–87	89	85–91
[45]		SPECT	80	71–87	85	79–90
[45]		FDG PET	90	84–94	89	81–94
[43]		^11^C-PiB	96	87–99	58 (median)	NA
[45]		CSF Aβ_42_	80	73–85	82	74–88
[45]		CSF T_tau_	82	76–87	90	86–893
[45]		CSF P_tau_	80	70–87	83	75–88
This analysis	^18^F-labelled tracer	Visual analysis	90	82–95	85	68–94
		Quantitative analysis	90	83–94	84	71–91

Therefore, based on the current evidence of diagnostic accuracy, the use of amyloid PET imaging cannot be advocated in preference to other existing diagnostic tests. There remains discussion over the most appropriate position (if any) of amyloid PET imaging in the clinical pathway. In 2011, the National Institute on Aging – Alzheimer’s Association workgroups on diagnostic guidelines for Alzheimer’s disease recommended updated criteria for the diagnosis of AD. These criteria do not include the use of any biomarker tests in the routine diagnostic process for AD, as the authors concluded that the core criteria provide sufficient diagnostic accuracy, and that more research is required, particularly regarding standardization and availability of the tests [6]. In 2014, the International Working Group for New Research Criteria for the Diagnosis of Alzheimer’s Disease proposed updated guidelines for the diagnosis of typical AD [1], updating their original 2007 criteria [46]. These criteria include in vivo evidence of AD pathology, requiring one of the CSF biomarker tests, amyloid PET imaging, or genetic tests, in addition to clinical criteria [1]. In 2012, the European Federation of Neurological Societies proposed guidelines on the use of neuroimaging in the diagnosis of dementia [47], which did not include the use of amyloid imaging in the routine clinical setting. They concluded that amyloid scans are likely to find clinical utility in patients with MCI, in patients with atypical symptoms, and for differentiating between AD and frontal-temporal lobe dementia [47]. However, the low specificity of beta-amyloid PET imaging in mixed populations of AD and MCI patients challenges this approach. Finally, in 2013, the Amyloid Imaging Task Force in association with the Society of Nuclear Medicine and Molecular imaging and the Alzheimer’s Association proposed appropriate use criteria for amyloid PET imaging [48]. They considered the use of amyloid PET imaging in a list of ten situations, with three considered an appropriate use of the technique: atypical presentation of AD, atypical age of onset and unexplained MCI [48]. Consensus agreement on the appropriate use of the techniques has not yet been reached.

There were limitations to our study. The analysis was carried out on reported data from the included studies, instead of data from individual patients. This would have added bias to the systematic review and meta-analysis as no accuracy checks could be carried out. Additionally, as is common with studies on imaging techniques that use ionizing radiation, the numbers of patients included in most studies was small.

### Conclusion

This systematic review and meta-analysis found no marked differences in the diagnostic accuracy of the three beta-amyloid radiotracers. All tracers perform better when used to discriminate between patients with AD and HC. The sensitivity and specificity for quantitative and visual analysis are comparable to those with other imaging or biomarker techniques used to diagnose AD. Further research is required to identify the combination of tests that provides the highest sensitivity and specificity, and to identify the most suitable position for the tracer in the clinical pathway.

## Electronic supplementary material

ESM 1 (DOCX 22 kb)
