# Supplementary material for: Diagnostic accuracy of 18F amyloid PET tracers for the diagnosis of Alzheimer’s disease: a systematic review and meta-analysis
Source: Eur J Nucl Med Mol Imaging. 2015 Nov 28;43:374–85. doi: 10.1007/s00259-015-3228-x (PMC4700091; doi:10.1007/s00259-015-3228-x)
Supplement: Supplementary file 1 — (DOCX 22 kb) [file 259_2015_3228_MOESM1_ESM.docx]

**Supplementary data for “Diagnostic accuracy of fluorine 18 amyloid PET tracers for the diagnosis of Alzheimer’s disease: systematic review and meta-analysis”**

| Table 1  Electronic Search Strategy for MEDLINE using the Ovid Interface | |
| --- | --- |
| 1. | exp dementia/ |
| 2. | alzheimer* disease.mp. |
| 3. | mild cognitive impairment.mp. |
| 4. | brain amyloid.mp. |
| 5. | 1 or 2 or 3 or 4 |
| 6. | amyloid imaging.mp. |
| 7. | amyloid tracer*.mp. |
| 8. | amyloid PET.mp. |
| 9. | amyloid positron emission tomography.mp. |
| 10. | florbetapir.mp. |
| 11. | AMYViD.mp. |
| 12. | Neuraceq.mp. |
| 13. | AV?45.mp. |
| 14. | flutemetamol.mp. |
| 15. | F-PiB.mp. |
| 16. | Pittsburgh compound B.mp. |
| 17. | PiB.mp. |
| 18. | florbetaben.mp. |
| 19. | BAY94?9172.mp. |
| 20. | AV?1.mp. |
| 21. | AZD4694.mp. |
| 22. | NAV4694.mp. |
| 23. | PET.mp. |
| 24. | Positron emission tomography/ or radionuclide imaging/ |
| 25. | 6 or 7 or 8 or 9 or 10 or 11 or 12 or 13 or 14 or 15 or 16 or 17 or 18 or 19 or 20 or 21 or 22 or 23 or 24 |
| 26. | clinical utility.mp. |
| 27. | management pathway.mp |
| 28. | sensitivity.mp. |
| 29. | specificity.mp. |
| 30. | positive predictive value.mp. |
| 31. | negative predictive value.mp. |
| 32. | outcome.mp. |
| 33. | 26 or 27 or 28 or 29 or 30 or 31 or 32 |
| 34. | 5 and 25 and 33 |
| 35. | limit 35 to (English language and human and yr="1990 -Current") |
| 36. | Deduplicate 35 |

| Supplementary data table 1: Modified QUADAS[^1^](#_ENREF_1)^,^ [^2^](#_ENREF_2) | | |
| --- | --- | --- |
|  | Original QUADAS | Modified QUADAS |
| 1 | Was the spectrum of patients representative of the patients who will receive the test in practice? | Participants in the study should be subjects with possible or probable Alzheimer’s disease (AD), other cognitive decline entities such as mild cognitive impairment (MCI), healthy controls above 55 years of age selected following a prospective patient inclusion. Details should have been provided about age distribution, female to male ratio and disease description as minimum information (patient characteristics table). |
| 2 | Were selection criteria clearly described? | A description of the selection criteria is considered sufficient when authors provide a clear definition of inclusion and exclusion criteria for study participation as well as period and location of recruitment. |
| 3 | Is the period between reference standard and index test short enough to be reasonably sure that the target condition did not change between the two tests? | A period of 4 weeks was chosen as a cut-off point; if studies performed the reference test and beta-amyloid scan within 4 weeks for more than 80% of the participants, score this item as ‘yes’. This item is scored ‘unclear’ when no information is given or when the time of either the reference or the index test is missing. |
| 4 | Did the whole sample, or a random selection of the sample, receive verification using a reference standard of diagnosis? | If it is clear from the study that all participants (or a random selection) who received a beta-amyloid scan also received verification of their disease status using the reference standard, then this item should be scored as ‘yes.' If some of the participants who received a beta-amyloid scan did not receive a verification of their disease state (or the selection was not random), then this item should be scored as ‘no.' If this information is not reported, this item should be scored as ‘unclear.' |
| 5 | Did patients receive the same reference standard regardless of the index test result? | In the case of beta-amyloid tracers if patients received the same reference test (biopsy or clinical diagnosis or other) regardless of the beta-amyloid uptake then score this item as ‘yes’. If patients with positive beta-amyloid scans received additional diagnostic tests score this item as ‘no’. |
| 6 | Was the execution of the index test described in sufficient detail to permit replication of the test? | To score as ‘yes’ the description of this item should include   - 1. Scanner model, injected dose, uptake period, emission time, attenuation correction method, reconstruction method, volume of interest delineation method.   2. If quantifiable results (SUVr) are reported then the use of a brain atlas template and/or an MRI, and appropriate reference region selection (cerebellar cortex) is mandatory to score this item as ‘yes.'   3. Measures to restrict motion or motion correction method |
| 7 | Was the execution of the reference standard described in sufficient detail to permit its replication? | To score as ’yes’ the description of this item should as a minimum include the specific guidelines (if any) under which clinical diagnosis was made, and whether or not specific neuropsychological tests were used. This should be provided either as a detailed description included in the manuscript or supplementary data or as a reference. |
| 8 | Were the index test results interpreted without knowledge of the results of the reference standard? | To confirm that authors were blinded to the results of the reference standard, a clear statement to this effect in the paper is required. If it is clear that no blinding occurred then score as ’no’. If no statement on blinding was given the item was scored ‘unclear’. |
| 9 | Were the reference standard results interpreted without knowledge of the results of the index test? | To confirm that authors were blinded to the results of the index test when interpreting the reference test, a statement to this effect in the text is required. If it is clear that no blinding occurred score as ‘no’. If no statement on blinding was given the item is scored as ‘unclear’. |
| 10 | Were the same clinical data available when test results were interpreted as would be available when the test is used in practice? | Clinical data are defined broadly to include any information relating to the participant that is obtained by direct observation, such as age, sex and symptoms. Knowledge of these factors can influence the visual analysis of PET data. Since at present the utility of these tracers in respect of specific pathways is unclear, this item was removed from the list. |
| 11 | Were withdrawals/missing values from the study explained? | If for any patients in the study either the reference or the index test was not reported but authors provided a valid explanation then score this item as ‘yes’. Otherwise score this item as ‘no’. |
| 12 | Is the reference standard likely to correctly classify the target condition? | The gold standard for establishing the diagnosis of AD is histopathology confirmation. However, clinical diagnosis based on appropriate international criteria is considered a valid alternative and is widely adopted. Score this item as ‘yes’ if either of the above has been used. |
| 13 | Was the reference standard independent of the index test (i.e. the index test did not form part of the reference standard)? | This item was removed from the list as the two tests are not related |
| 14 | Were uninterpretable/intermediate test results reported? | If it is clear what happened to all participants who entered the study, or a flow diagram of study participants is given, score as ‘yes’. If no withdrawals are reported and all results are available for all participants, then score as ‘yes’. If some of the participants who entered the study did not complete it and these haven’t been accounted for score as ‘no’. If it is not clear whether all participants who entered the study were accounted for, then score as ‘unclear’. |

References:

**1.** Whiting P, Rutjes A, Reitsma J, Bossuyt P, Kleijnen J. The development of QUADAS: a tool for the quality assessment of studies of diagnostic accuracy included in systematic reviews. *BMC Medical Research Methodology.* 2003;3(1):25.

**2.** Chalkidou A, Landau DB, Odell EW, Cornelius VR, O'Doherty MJ, Marsden PK. Correlation between Ki-67 immunohistochemistry and 18F-fluorothymidine uptake in patients with cancer: A systematic review and meta-analysis. *European journal of cancer.* Dec 2012;48(18):3499-13.
